# PHYSICAL ACTIVITY, QUALITY OF LIFE AND BODY IMAGE OF CANDIDATES TO BARIATRIC SURGERY

**DOI:** 10.1590/0102-672020180001e1349

**Published:** 2018-06-21

**Authors:** Bruno Leandro de Melo BARRETO, Jones Silva LIMA, Diogo Barbosa de ALBUQUERQUE, Flavio KREIMER, Álvaro Antonio Bandeira FERRAZ, Josemberg Marins CAMPOS

**Affiliations:** 1 Department of Surgery, Universidade Federal de Pernambuco; 2 Associated Postgraduate Program in Physical Education, Universidade de Pernambuco/Universidade Federal da Paraíba, Recife, PE, Brazil.

**Keywords:** Obesity, Physical activity, Quality of life, Body image., Obesidade, Atividade física, Qualidade de vida, Imagem corporal.

## Abstract

**Background:**

Physical activity enhances quality of life and body image in obese. Behavioural changes are useful tools to increase life conditions of this population.

**Aim:**

To evaluate the physical activity level of candidates to bariatric surgery and its relation with quality of life and body image, when patients are encouraged weekly by personal trainers.

^)^ **Method:** This is a prospective, interventional and longitudinal study with quantitative analysis. Patients were divided into two groups, control (n=28) and interventional (n= 10). Both groups received physical activity and nutritional recommendations and psychological support. Were used the SF36 and Body Shape questionnaires to assess physical

activity level and body image and pedometers to count weekly steps. Patients were followed during 12 weeks.

^)^ **Results:** Were found significant difference in the domains physical activity (p=0.019), pain (p=0.0001) and health general status (p=0.021). No significant difference in body weight (p=0.095) was noted.

**Conclusion:**

When assisted by personal trainers, obese patients can change behavior, increase health quality and physical activity levels and experience less pain. Increase in physical activity, when well structured can benefit these patients.

## RESUMO

**Racional:**

A atividade física influencia na qualidade de vida e imagem corporal dos obesos. A possibilidade de mudança nos hábitos de vida é ferramenta para melhoria desta condição.

**Objetivo:**

Avaliar o nível de atividade física dos pacientes e sua relação com a qualidade de vida e imagem corporal, quando incentivado semanalmente por profissional de educação física.

**Métodos:**

Estudo prospectivo, intervencional do tipo longitudinal com abordagem quantitativa. Os pacientes foram distribuídos em dois grupos, controle (n=28) e intervencional (n=10). Ambos receberam recomendações relacionadas a atividade física, apoio psicológico e recomendações nutricionais. Foi utilizado os questionários SF36 e o questionário Body Shape para mensurar essas variáveis e no grupo intervencional a utilização de um pedômetro. O estudo durou 12 semanas.

**Resultados:**

Através do SF-36 foi observada diferença no nível de atividade física entre o grupo experimental após os três meses. Houve diferença significativa nos domínios AF (atividade física, p=0,019), dor (p=0,0001) e estado geral de saúde (p=0,021). Não existiu diferença significante no peso corporal (p=0,095).

**Conclusão:**

Quando assistido por profissional de educação física, o paciente obeso muda os hábitos, melhora na qualidade de vida e sente menos dor; o aumento no nível de atividade física, quando bem estruturado, pode trazer benefícios ao seus praticantes.

## INTRODUCTION

Obesity highly impacts psychological disorders, including depression, eating disorders, distorted body image and low self-esteem. Factors such as depression and anxiety are three to four times more prevalent in obese, who still suffer from prejudice and social discrimination^4^.

Physical activity is one of the most important factors for acquiring good quality of life and improving body image being recommended in the pre and postoperative time, improving mobility and cardiorespiratory capacity in obese patients^**5**^ ^**,**^ ^**11**^ ^**,**^ ^**13**^. Regular practice of physical activity can promote improvement in cardiorespiratory and physical capacities, cognitive functions (memory, attention, reasoning), body mass control, and reduction of depression, anxiety and stress^6^ ^,^ ^10^.

Bariatric surgery is considered a tool for controlling severe obesity, with resolution of comorbid conditions associated with weight gain and metabolic disorders^3^ ^,^ ^8^ ^,^ ^9^.

This study aimed to assess the influence of weekly incentive of physical education specialist in the level of physical activity and its relation with quality of life and body image in obese patients.

## METHODS

This is a prospective, interventional, longitudinal study with a quantitative approach. The project was approved by the Research Ethics Committee of the Health Sciences Center of the Federal University of Pernambuco - No. 915.390/2014 - CAAE 43279115.4.0000.5208. Candidates for bariatric surgery at the Hospital das Clínicas of the Federal University of Pernambuco, Recife, PE, Brazil, were invited to participate of the project from June 1, 2015 to June 1, 2016.

Initially, all the patients received orientation regarding physical activity, and then were divided into two groups. The control group (n=28, 22 female) received nutritional orientation, psychological counseling, guidance on physical activity and a pedometer to record the number of weekly steps. The experimental group (n=10, all female) received nutritional counseling, psychological counseling, physical activity guidelines and a pedometer to record the number of weekly steps and was also followed weekly by a physical education professional. Yamax® SW-700 Digi-Walker pedometers were used. Patients in the control group were weighed monthly and the experimental groups were weekly. The patients completed the SF-36 and Body Shape questionnaires at baseline and after three months. 

### Statistical analysis

The experimental variables were submitted to the Shapiro-Wilk normality test; the statistical hypothesis tests were those of non-parametric nature.

## RESULTS

Responses to the questionnaire SF36 (Table 1) showed differences in functional capacity (p=0.018) and physical activity (p=0.019) between the groups after three months; there was also improvement in the domains body pain (p=0.0001), general health perceptions (p=0.021) and vitality (p=0.005). In this study, we did not find a significant improvement in the domains social role functioning (p=0.076), emotional role functioning (p=0.095) and mental health (p=0.076). Our results showed no significant difference in body weight (p=0.095, Table 2) and body image evaluated by the Body Shape questionnaire (Table 3, p=0.125). We found a significant difference in the quality of life of those who had weekly follow-up and increased the level of physical activity (Table 4, p=0.0001). The incentive of the experimental group resulted in a significant difference in the number of weekly steps (Table 5, p=0.0001).

**TABLE 1:** SF-36 Questionnaire responses

SF-36 domains	Before		p	After		p
	_X	SD		_ X	SD	
Physical role functioning						
Control group	32.1	21.8	p=0.935	40.5	21.8	p=0.018
Interventional group	29.5	14.2		65	27.4	
Physical functioning						
Control group	36.6	39.4	p=0.757	22.3	26.6	p=0.019
Interventional group	30.0	35.0		55	38.7	
Body pain						
Control group	44.1	26	p=0.182	33.1	19.7	p=0.0001
Interventional group	33	16.1		60.5	18.9	
General health perceptions						
Control group	39.5	14.1	p=0.807	37.2	18.4	p=0.021
Interventional group	42.9	10.9		52.9	14.4	
Vitality						
Control group	41.4	19.5	p=0.613	46.8	17.7	p=0.005
Interventional group	37	18.7		62.5	8.6	
Social role functioning						
Control group	56.3	29.6	p=0.883	54.5	25.3	p=0.076
Interventional group	57.5	29.6		71.3	22.9	
Emotional role functioning						
Control group	39.3	42.6	p=0.503	36.9	43.8	p=0.095
Interventional group	30	48.3		66.7	44.4	
Mental health						
Control group	56.1	22.7	p=0.082	51.9	22.7	p=0.076
Interventional group	44.8	14.2		65.6	15.1	

**TABLE 2:** Body weight

Body weight (kg)	Before			After		
	Mean	SD		Mean	SD	
Control group	120	24.7	p=0.423	119.9	22.5	p=0.095
Interventional group	113.8	14.5		108.8	14.1	

**TABLE 3:** Body Image analysis using Body Shape

Body image (Body Shape)	Before			After		
	Mean	SD		Mean	SD	
Control group	137.3	35.5	p=0.286	146.9	32.4	p=0.125
Interventional group	123.4	27.7		126.9	31.8	

**TABLE 4:** Quality of Life SF-36

Quality of Life	Before			After		
	Mean	SD		Mean	SD	
Control group	83.2	17.1	p=0.23	82.5	15.3	p=0.0001
Interventional group	78.1	9.2		103.4	14.4	

**TABLE 5:** Number of weekly steps (x1000)

Weekly steps (x1000)	Before			After		
	Mean	SD		Mean	SD	
Control group	14.2	2.1	p=0.0001	15.8	2.1	p=0.0001
Interventional group	18.3	3.2		24.6	5.8	

## DISCUSSION

The relation between the purposed level of physical activity and the actual performance was determined by daily activity recommendations and evaluated adopting the SF-36 questionnaire and a pedometer to each patient, with goals in the ranges of 3-5 thousand steps, 5-8 thousand steps and 8-10 thousand steps daily, considering the motivation and social status in accessing public places for physical activity. Even with personal trainer incentive, the number of steps did not achieve the goals established by the project and international recommendations. Even though, their improvement was enough to impact quality of life, body pain and general health status domains.

Despite of the positive results of the domain physical functioning in the SF-36, when a pedometer was used, we identified low levels of physical activity, suggesting the questionnaire was inadequate for this population.

Our findings regarding physical activity level, number of steps, weight and weight loss differ from a study carried out during 16 weeks with 51 overweighted adult women (BMI ≥25 kg/m²)^7^, that included supervised physical activity three times a week and health education once a week; the researchers observed a significant difference in weight and BMI. A research held in the US with 199 subjects found active patients presented better weight reduction, mental health, quality of life and self-esteem^8^.

Another study conducted in 20011^9^ which evaluated 455 adult subjects undergoing Bariatric Surgery showed similar results in the domain body pain; the patients received an activity monitor that recorded the steps/minute rate, and an exercise diary before and one year after the surgery.

We found a greater adherence of women, in view of their greater concern with body aesthetics. However, in this study the level of physical activity was not related to changes in body image. Women and individuals with higher BMI presented greater body dissatisfaction, agreeing with the result aof other authors^10^.

Adherence to physical activity programs and change in eating habits were higher in female gender. The interventional group was more motivated in relation to remaining in the multidisciplinary monitoring program and did not give up the surgery.

Our research presented some limitations related to the method. The forms were completed in two steps, due to their filling during the waiting for medical consultations. Another challenge was the resistance of patients to participate when informed that the project will involve physical activity practice, decreasing the sample. Some questionnaires were collected unanswered, strikethrough or filled in an unsecured manner, due to difficult understanding or lack of interest.

Measuring patients’ body composition would have increased the quality of our study; however, difficulties of accessing DEXA or Bioimpedance were faced due to financial and structural limitation, in view of the service demand.

No direct relationship was found between the level of physical activity using the SF-36 questionnaire in domain physical functioning and the weekly number of steps. Therefore, future studies should use the pedometer instead of questionnaires to measure this variable. In this population, it was not possible to identify changes in the body image when related to the level of physical activity.

## CONCLUSION

When assisted by a physical education specialist, people change their habits, improve their quality of life, and feel less pain. Increasing the level of physical activity, when well structured, can bring benefits to the patients.
